# Recreational Nitrous Oxide-Induced Subacute Combined Degeneration of the Spinal Cord

**DOI:** 10.7759/cureus.19377

**Published:** 2021-11-08

**Authors:** Priyal Agarwal, Si Yuan Khor, Steven Do, Lawrenshey Charles, Richa Tikaria

**Affiliations:** 1 Internal Medicine, Michigan State University, Lansing, USA; 2 Infectious Disease, Michigan State University, Lansing, USA

**Keywords:** vitamin b12 deficiency, inhaled nitrous oxide, subacute combined degeneration, whip it, rare cause of vitamin b12 deficiency, laughing gas, whippet, functional vitamin b12 deficiency

## Abstract

There is rising use of recreational nitrous oxide (N₂O) in the community because of its availability as “whippet” canisters. Nitrous oxide use is still legal and outside the purview of the Drug Enforcement Administration (DEA). It is not detected on a routine drug screen, and patient history is key to establishing the diagnosis. We highlight a case of subacute combined degeneration in a young patient secondary to recreational nitrous oxide use, which improved with vitamin B12 replacement. A 19-year-old male with a history of recreational nitrous oxide use presented with progressive bilateral lower extremity paresthesia and ataxia. Neurological examination revealed deficits in vibration and proprioception, motor weakness, and diminished reflexes in the bilateral lower extremities. The laboratory results were significant for pancytopenia, profound vitamin B12 deficiency (55 ng/mL), and elevated methylmalonic acid (2.14 umol/L). The urine drug screen was negative. MRI showed subacute degeneration of the spinal cord dorsal column at C2-C5. Treatment with intramuscular cyanocobalamin resulted in the normalization of pancytopenia and B12 levels (573 ng/mL). The patient had partial resolution of neurological symptoms following the initiation of parenteral vitamin B12 replacement. The mechanism of subacute combined degeneration in the setting of nitrous oxide toxicity appears to be mediated by functional B12 deficiency. Oxidation of cobalt ion of vitamin B12 by nitrous oxide renders it unavailable as a coenzyme, leading to the accumulation of by-products that enter lipid metabolism, resulting in abnormal myelin synthesis, which ultimately manifests as subacute combined degeneration. Vitamin B12 deficiency of unclear etiology should raise suspicion for nitrous oxide toxicity as early initiation of replacement therapy with vitamin B12 can improve neurological function.

## Introduction

The recreational use of nitrous oxide (N₂O) or laughing gas continues to increase in the community [1,2]. It is a cheap, readily available, euphoria-inducing agent undetectable on routine drug screens. Although previously thought to be safe, we now know that nitrous oxide has short- and long-term adverse effects on multiple systems, including hematological, immune, neurological, and reproductive [3]. We present a case of myelopathy in a young patient secondary to recreational nitrous oxide use.

## Case presentation

A healthy 19-year-old male presented with a one-week history of progressive bilateral lower extremity paresthesia and ataxia. The patient admitted to inhaling 100-150 nitrous oxide cartridges weekly for the past year for recreational use. Other past medical history or family history was unremarkable. He denied any other drug use. General physical examination was unremarkable. Neurological examination revealed mild motor weakness (4/5), deficits in vibration and proprioception sensation (in a length-dependent manner), and diminished deep tendon reflexes (1+) in bilateral lower extremities. The patient reported a subjective loss of sensation, but on testing, pain and temperature sensations were intact in both lower extremities. Romberg’s test was positive, and his gait was ataxic. The rest of the neurological examination, including tests for cognition, cranial nerves, and coordination, was normal. Babinski and Hoffmann’s reflexes were negative. Bilateral upper extremity showed normal strength, sensations, and reflexes.

Laboratory testing revealed the following: pancytopenia with normocytic anemia (hemoglobin, 7.5 g/dL [reference range: 12.5-15.5 g/dL]; mean corpuscular volume, 92 fL [reference range: 81.6-98.3 fL]); white blood cell count, 3.3 × 10^9^/L [reference range: 4-12 × 10^9^/L]; and platelet count, 100 × 10^9^/L [reference range: 150-400 × 10^9^/L]), elevated lactate dehydrogenase (LDH, 536 U/L [reference range: 100-350 U/L]), severe vitamin B12 deficiency (vitamin B12, 55 ng/L [reference range: 211-911 ng/L]), and elevated methylmalonic acid (methylmalonic acid, 2.14 umol/L [reference range: ≤0.4 umol/L]). Peripheral smear showed normocytic anemia with few macrocytes and leukopenia. Urine drug screen was negative.

**Figure 1:**
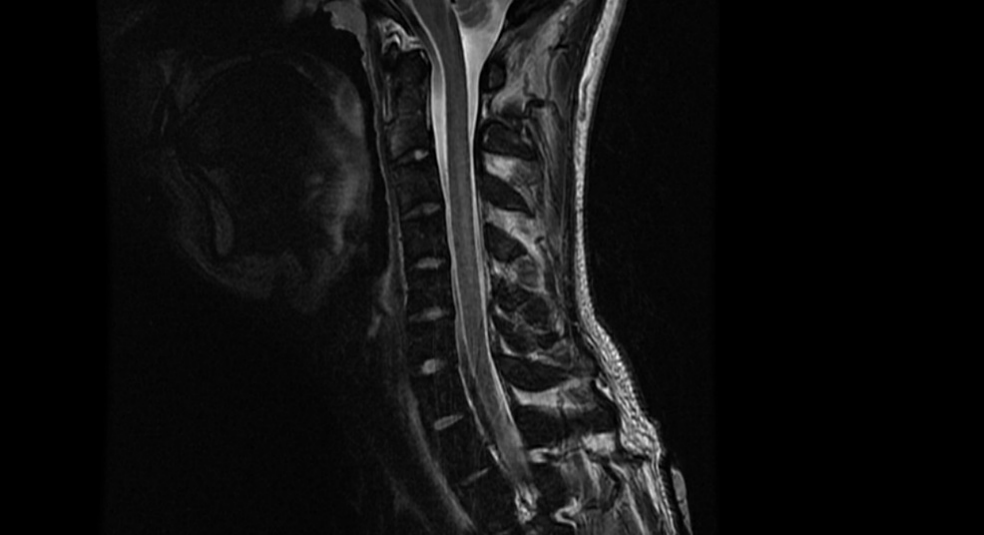
MRI T2-weighted sagittal view of the cervical spine showing increased T2 signal within the dorsal aspect of the cervical spinal cord from C2 to C5

MRI cervical spine showed subacute degeneration of the spinal cord dorsal column at the C2-C5 level (Figure 1). The patient was started on treatment with intramuscular cyanocobalamin. We provided education and counseling regarding the adverse effects of nitrous oxide use, and the patient expressed motivation not to use nitrous oxide in the future. The follow-up laboratory results showed normalization of pancytopenia and vitamin B12 levels (573 ng/mL). The patient had partial resolution of neurological symptoms during hospitalization, and he was discharged with a plan to continue outpatient physical rehabilitation.

## Discussion

Nitrous oxide (N₂O) is a colorless, tasteless, and odorless gas with relaxant and euphoric properties [4]. Nitrous oxide is cheap, readily available, and undetectable on a routine drug screen. Nitrous oxide is readily available as "whippet" canisters used to fill balloons or whipped cream dispensers [1]. It is usually inhaled from balloons or the dispenser for recreational purposes.

The recreational use of nitrous oxide has been increasing and poses serious health risks with long-term neurological sequelae. According to the global drug survey of 2018, nitrous oxide is the seventh most popular drug worldwide, excluding alcohol, nicotine, and caffeine [5].

The mechanism of subacute combined degeneration in the setting of nitrous oxide toxicity appears to be mediated by functional B12 deficiency, which ultimately results in decreased normal myelin synthesis. 5-Deoxyadenosylcobalamin, a metabolite of cobalamin, is a coenzyme in the reaction that catalyzes the conversion of methylmalonyl CoA to succinyl CoA, which subsequently enters the citric acid cycle. Nitrous oxide oxidizes the cobalt ion of vitamin B12, which irreversibly inactivates cobalamin. Inactivated cobalamin is unable to function as a coenzyme. Methylmalonyl CoA is accumulated, which enters into the lipid pathway and results in the incorporation of abnormal fatty acids into neuronal lipids. This results in decreased myelination of the lateral and posterior columns of the spinal cord or subacute combined degeneration [6-8].

Subacute combined degeneration of the spinal cord is a neurodegenerative disorder associated with degeneration of the dorsal and lateral white matter of the spinal cord [9]. Patients usually present with ascending paresthesia, weakness, ataxia, and loss of sphincter control. It is most commonly associated with vitamin B12 deficiency. MRI shows symmetric increased T2 signal in the posterior and lateral columns of the cervical and thoracic spinal cord [10].

When there is suspicion of subacute combined degeneration in a patient with normal vitamin B12 levels, it is recommended to measure serum methylmalonic acid and homocysteine, which will be elevated.

Early recognition and initiation of replacement therapy with vitamin B12 can result in the improvement of neurological function in nitrous oxide-induced vitamin B12 deficiency [11].

## Conclusions

Vitamin B12 deficiency of unclear etiology should raise suspicion for nitrous oxide toxicity. History of nitrous oxide use is key to establishing the diagnosis and should be elicited from the patient. In the presence of neurological symptoms and history of nitrous oxide use, if vitamin B12 levels are normal, serum methylmalonic acid and homocysteine levels should be measured to establish the diagnosis. Early treatment with vitamin B12 replacement therapy can result in the improvement of neurological function in nitrous oxide-induced vitamin B12 deficiency, as seen in our patient.
